# Integrated approaches to malaria control - addressing new challenges to malaria research

**DOI:** 10.1186/1475-2875-11-S1-P104

**Published:** 2012-10-15

**Authors:** Martin Wiese

**Affiliations:** 1 International Development Research Center, IDRC, Ottawa, Ontario, Canada, K1G 3H9

## 

While significant progress in understanding and controlling malaria has been made, extensive regions of sub-Saharan Africa remain hotspots of transmission, burden and impacts from malaria [1]. The pathogenicity of falciparum malaria and the high vectorial capacities of *Anopheles gambiae* vector systems compound in Africa with poor and unsecured livelihoods, weak health and disease surveillance systems, and agro-ecological transformations on an almost continental scale [2]. Moreover, the patterns of risks and vulnerabilities to malaria remain dynamic under global and climate changes.

Today’s efforts against malaria rely on innovation of tools and funding mechanisms, and the progressive scaling up of intervention packages especially targeting the elimination of malaria at its current transmission limits to ‘shrink the malaria map’ [3]. However, delivery modalities for intervention packages are challenged by poor, remote and unstable conditions and weak health systems. Thus, new approaches are needed for developing, delivering and maintaining malaria control in areas where high or unstable disease transmission compounded with systemic vulnerabilities cause the world’s most significant malaria burden [4-6].

‘Synergy harvesting’ should open new options to tackle the root causes of malaria risks and vulnerabilities linked to health systems, livelihoods, and ecosystems conditions. Such integrated research will require collaboration of researchers across sectors and disciplines. However, we currently lack evidence on the added values of integrated malaria research and control from combined investments into health systems strengthening, livelihoods improvements and, for example, agro-ecosystems interventions.

The rationale for a new initiative dedicated to develop integrated research partnerships on malaria in Africa stems from the need for a consolidated evidence base, enhanced capacities, and stronger advocacy for outcome oriented malaria research that transcends disciplinary and sectorial boundaries [7].

Integrated research requires systems thinking, outcome-oriented monitoring and evaluation approaches, collaboration - across disciplines, sectors and regions -, multi-stakeholder engagement, and sensitivity to social equity. The complexity of these challenges currently outpaces the existing capacities of research teams, especially in regions most affected by malaria. These same regions also have commonly weak research capacities and compartmented institutional landscapes. With this in mind and based on four decades of investments into applied malaria research in developing regions across the globe, Canada’s International Research Development Centre, IDRC, is currently supporting the formation and consolidation of multi-national, multi-disciplinary research consortia in high-transmission and high-burden countries of Sub-Saharan Africa. Since 2011, 3 multi-country consortia, selected through an open competitive process, mobilize early-career researchers from research institutions of 10 countries in this region (Benin, Burkina Faso, Cameroun, Kenya, Mali, Niger, Rwanda, Tanzania, Togo, Uganda). Teams develop research, build collaboration and alliances, advocate and leverage support for integrated malaria research and control. They seek to identify and assess synergy opportunities for malaria control from investments into environmental, livelihoods and health systems improvements.
